# Non-participation in mammographic screening – experiences of women from a region in Sweden

**DOI:** 10.1186/s12889-020-8306-8

**Published:** 2020-02-12

**Authors:** Maria Norfjord van Zyl, Sharareh Akhavan, Per Tillgren, Margareta Asp

**Affiliations:** 1 Division of Public Health Sciences, School of Health, Care and Social Welfare, Mälardalen University, Box 883, 721 23 Västerås, Sweden; 2 Division of Caring Sciences and Health Care Pedagogics, School of Health, Care and Social Welfare, Mälardalen University, Box 883, 721 23 Västerås, Sweden

**Keywords:** Experiences, Mammographic screening, Non-participation, Perceptions, Public health

## Abstract

**Background:**

Understanding women’s life conditions regarding their non-participation in different health-promoting and disease-preventing activities is important as it may draw attention to potential areas for improvement in the healthcare sector. Mammographic screening, a disease-preventing service, facilitates early detection of any potential malignancies and consequently prompts initiation of treatment. The reasons for non-participation in mammographic screening can be understood from different perspectives, such as socioeconomic and lifestyle-related determinants of health. This study aims to gain a deeper understanding of women’s experiences and perceptions about non-participation in mammographic screening in a Swedish region with a single mammographic facility.

**Methods:**

Data from individual semi-structured interviews, conducted in 2018 with eleven women between the ages of 48 and 73, were analysed by a qualitative content analysis.

**Results:**

The findings reveal three main categories: 1) doubts regarding mammographic screening and its organisation, 2) sense and sensibility in the decision to refrain from mammographic screening, and 3) dependency and options. These three categories indicate aspects, such as the individual’s life situation, accessibility to the offered service, and the flexibility of the healthcare system, that need to be considered to improve the organisation of mammographic screening.

**Conclusion:**

Listening to the women’s voices regarding their experiences and perceptions about mammographic screening is important as individual characteristics and social circumstances interact with healthcare and affect the degree of participation.

## Background

Good health facilitates individual development [1], as well as a country’s socioeconomic growth [2]. Access to healthcare services is a factor that contributes to good health, and the actual use of healthcare services is important, not merely the presence of a facility [3]. One healthcare service that is represented in all Swedish regions is the population-based mammographic screening programme that every 2 years invites all nationally registered, 40–74-year-old women to undergo a breast check-up [4]. The mammographic screening has been free of charge since June 2016. The screening facilitates early detection of any potential malignancies [5–7], but the benefits of the screening in reducing the breast cancer mortality rate have been debated, for instance, versus the risk of overdiagnosis [8], which refers to the detection of breast cancer at mammographic screening that would not otherwise have been found clinically in the woman’s lifetime [9]. In Sweden, as in many other countries, the benefits of the screening have been regarded as outweighing the risk [4, 10]. Offered as a provider-led systematic programme [11], it is initiated by the state as a strategically proactive measure for cancer care [12], and a high participation rate contributes to the cost effectiveness of a screening programme [6].

The use of mammographic screening or the rate of participation in it can be understood from different perspectives, such as socioeconomic and lifestyle-related determinants of health [13]. The reasons for non-participation may be distance [14–17], beliefs, fear of cancer [18], negative experiences regarding healthcare or encounters with the staff, pain during the procedure [19–22], practical issues regarding accepting the invitation to undergo breast screening [18], and inconsideration for women’s life situation related to the rigidity of the healthcare system [21]. In Sweden, approximately 20% of 40–74-year-old women declined the offer to be screened, and the non-participation rate in the Swedish counties varied between 12 and 28% in 2015. Assessment regarding non-participation is needed at the regional level since some determinants are influenced by the resource allocation and the decisions made at that level [23]. Thus, this study contributes to a richer understanding of the reasoning behind the decision to refrain from mammographic screening from a qualitative perspective.

This study aims to gain a deeper understanding of women’s experiences and perceptions about non-participation in mammographic screening in a Swedish region with only one mammographic facility.

## Materials and methods

Based on a descriptive design, individual semi-structured interviews [24] were conducted, and a content analysis of the data was performed.

### Setting

The region is in Mid-Sweden, with 273,929 inhabitants, just below the median of 286,547 in Sweden’s 21 regions, and a female population of 135,888, also slightly below the median of 141,947 for the female population in Sweden’s regions [25]. The region has been chosen because, to the best of the authors’ knowledge, studies at the regional level have mainly been conducted in Sweden’s three metropolitan areas. A regional perspective is also important for other areas in Sweden, such as the one under study, because the responsibility for health care rest on the regions [26]. Additionally, this region has only one stationary screening facility as a service point; the women’s experiences are based on visiting the same screening facility.

The mammographic screening facility is situated in the region’s only median-sized town [23], and the non-participation rate was 16% in 2015 [27]. Until 2006, a mobile mammographic unit and the stationary screening facility simultaneously served the women residing in the region’s other municipalities, defined as commuting municipalities near medium-sized towns and commuting municipalities near small towns [23], situated ~ 20–80 km from the stationary screening facility [14]. The mobile unit was then taken out of service, due to the shift from analogue to digital mammographic screening technology, and was not replaced with a new mobile unit.

Every 2 years a letter of invitation to mammographic screening is sent to the women. The letter contains information, including the appointment schedule (date and time), the purpose of the screening, how the screening is done, possibilities to re-schedule the appointment, the voluntary participation, links to further information (such as information in other languages about mammographic screening) and contact details of the mammographic screening facility. To be invited, a woman must have a postal address. If the woman has contacted the facility in order to not be invited at all, she will be excluded from further invitations. This status can thereafter be reconsidered at her discretion.

### Selection of informants

To identify the women who had declined the mammographic screening, the administrative register “Radiological Information System” at the local mammographic screening facility was used. The register contains information about invitation dates, participation and non-participation. If the woman has attended, the letter “D” is noted in the register and stands for *deltagit* (the Swedish word for attended). The letter “A” denotes “actively passive” (*aktivt passiv* in Swedish), which indicates that the woman has actively contacted the mammographic facility in order to decline attendance. The letter “P” signifies “passive” (*passiv* in Swedish), meaning that the woman has never contacted the facility to decline the offer and has never shown up at the appointed time.

The staff at the screening facility conducted a systematic sampling [28]. From a total of 4355 non-participating women in the chosen region, 200 women were selected for the sample. The choice of 200 women was based on a previous Swedish study with a sample of 187 women [29]. To ensure that the sample would be as representative as possible for a calendar year, an even distribution across the months was made. The inclusion criteria were 40–74-year-old women who were residents of the region, fluent in Swedish and had declined the two most recent invitations to mammographic screening from 2016 and earlier (Table 1). A letter and an interest request were mailed to the selected women, with the first author’s contact information if further details were needed. A follow-up of the interest request was mailed after 4 weeks, resulting in a total of 11 informants. They decided where the interviews should take place, as follows: at home (*n* = 6), at the university (*n* = 3), at work (*n* = 1) and over the telephone (*n* = 1). The first author conducted the interviews.

**Table 1:** Socio-demographic characteristics of the informants

	Distance (km) from reference city (location of mammographic facility)	Age (median age = 63 years)	Marital status	Level of education	Employment status	Country of origin	Active passive (A), Passive non-participation (P) or a combination for the two last invitations
Informant 1	60–79	68	Cohabiting/Married	Tertiary	Self-employed	Sweden	A, A
Informant 2	40–59	63	Cohabiting/Married	Tertiary	Teacher	Sweden	A, A
Informant 3	< 19	61	Single	Primary	On disability pension	Sweden	A, A
Informant 4	40–59	68	Divorced/ Widowed	Tertiary	Pensioner	Sweden	A, P
Informant 5	< 19	64	Cohabiting/Married	Tertiary	Accountant	Sweden	P, P
Informant 6	< 19	59	Cohabiting/Married	Tertiary	Physiotherapist	Sweden	A, A
Informant 7	40–59	62	Divorced/ Widowed	Primary	Unemployed (Housewife)	Lebanon	A, A
Informant 8	20–39	58	Cohabiting/Married	Tertiary	Teacher	Sweden	A, A
Informant 9	40–59	65	Divorced/ Widowed	Secondary	Nursing assistant	Sweden	P, A
Informant 10	60–79	48	Cohabiting/Married	Tertiary	Artist	Sweden	A, A
Informant 11	60–79	73	Divorced/ Widowed	Tertiary	Consultant /Pensioner	Sweden	A, A

Note. Distance from reference city [14]

Eleven women (Informant 1–Informant 11), whose ages ranged from 48 to 73 years (mean age = 62. 6 years), agreed to be interviewed. Two women had a primary-level education, one attained the secondary level, and the remaining eight women had a tertiary-level education. The women’s employment status and positions were as follows: recipient of a disability pension (*n* = 1), pensioner (*n* = 1), partly a pensioner and partly working as a consultant (*n* = 1), unemployed (*n* = 1), self-employed (*n* = 1), accountant (*n* = 1), teacher (*n* = 2), physiotherapist (*n* = 1), nursing assistant (*n* = 1) and artist (*n* = 1). All the women had undergone mammographic screening at least once before the two most recent invitations (Table 1).

### Data collection

The interviews were conducted by using a semi-structured interview guide [30], after a written and signed informed consent form was obtained from all study participants. The following questions are two examples from the guide: “How did you reason around your decision to refrain from mammographic screening?” “What factors have influenced your decision to refrain?” (Additional file 1: Interview Guide). Depending on the answers, further questions followed to obtain rich descriptions from the informants. Short notes were made during the interviews to summarise what had been said and to allow the informants to correct or elaborate on their answers. Ranging from 23 to 58 min each, the interviews were audiotaped and transcribed verbatim by the first author.

### Data analysis

A content analysis with an inductive approach [31] was performed. Each interview was initially read twice and reread during the analysis process, which entailed going back to the data material to ensure that no relevant units of the analysis had been overlooked. In each interview, meaning units (text related to the aim) were highlighted, followed by an open coding that described the content of the units by making comments on the margins. Short notes were made if any additional reflections presented themselves during the analysis (Table 2).

**Table 2:** Examples of the analysis

Meaning unit	Code	Subcategory	Generic category	Main category
It is more like…then one mentally “okay, five minutes more I have to endure” … (Informant 10)	Handling of pain during mammogram	Approaches related to mammogram	Physical and psychological tensions	Sense and sensibility in the decision to refrain
And I also think that if there is anything there, I [would] rather not know. (Informant 4)	Avoidance of knowledge	Unmanageable pain and knowledge		
We don’t have it [breast cancer] in the family. (Informant 6)	Hereditary risk	Trust in self-awareness of the body and risk assessment	Rationalisation when deciding to refrain from mammographic screening	
But if I have a group waiting for me, [I would rather refrain then] … (Informant 2)	Prioritisation of others	Diminishing of own needs		

After the same procedure was followed for each interview, all the codes were transferred into a coding sheet and thereafter grouped and categorised by comparing the groupings and determining their commonalities and differences. The same process occurred as the abstraction process continued with the creation of generic categories and finally, the main categories [31]. During the analysis process, the last author read through the first author’s interpretation of the data, and creation of both generic and main categories. This process strengthened the study’s credibility because consensus was reached. Additionally, the consolidated criteria for reporting qualitative studies (COREQ) were respected [32].

## Results

The findings from the analysis resulted in three main categories: 1) doubts regarding mammographic screening and its organisation, 2) sense and sensibility in the decision to refrain from mammographic screening, and 3) dependency and options. These were derived from six generic categories and 12 subcategories (Table 3). Quotes from the informants (Informant) are used to illustrate the main categories. After each quote, a number in parentheses is assigned to each informant in order to distinguish them from one another. An ellipsis (…) indicates omitted words or sentences. In any part of an excerpt where the author [A] comments or adds a clarifying word/phrase or the informant pauses or hesitates, the text is enclosed in square brackets.

**Table 3:** Main categories, generic categories and subcategories

Main categories	Generic categories	Subcategories
Doubts regarding mammographic screening and its organisation	Lack of trust in mammographic screening equipment and its arrangements	Different mammographic screening possibilities
		Secure and reliable mammogram and apparatus
	Ambivalent appreciation for mammographic screening	Appreciation for benefit and purpose
		Personal and professional treatment
Sense and sensibility in the decision to refrain	Physical and psychological tensions	Approaches related to mammogram
		Unmanageable pain and knowledge
	Rationalisation when deciding to refrain from mammographic screening	Trust in self-awareness of the body and risk assessment
		Diminishing of own needs
Dependency and options	Importance of flexible and individually adapted systems and solutions	Service-adapted operation and local representation
		Unsatisfying individual solutions and degree of participation
	Insufficient possibilities to influence external circumstances and the women’s own priorities	Resource demands in relation to value
		Strenuous distance and transports for attendance

### Doubts regarding mammographic screening and its organisation

Doubts refer to the type of technology used for the mammographic screening and its service delivery, which manifest themselves in two generic categories: 1) lack of trust in the mammographic screening equipment and its arrangements and 2) ambivalent appreciation for mammographic screening.

#### Lack of trust in mammographic screening equipment and its arrangements

This perception is expressed in relation to different mammographic screening possibilities and unnecessary suffering. It is understood that the screening procedure may be less painful with another type of machine, but the agents in charge of procuring the mammographic equipment in the region prioritise the economy over the women’s experiences during the mammogram by using the existing equipment.

*… I do understand that it is probably about economy. That one [the Health Care Organisation] has invested in those horrible machines, ehhm, and therefore wants to use them… as it is set up... in this region at least.* (Informant 10).

The accuracy of the screening is also mentioned as a reason to refrain from the screening.

*… I have also read that one can do this type of examination [mammogram], and then I still can have something [abnormalities] just a week after that one [the screening apparatus] didn’t see anything.* (Informant 8).

The questioning of the organisation’s decision regarding the phase-out of the mobile mammographic unit is also highlighted. The mobile unit facilitated the women’s access to a mammogram in the municipality where they resided. Some of the women raise the question of why the information and the motives underlying the decision have not been clearly articulated, and no dialogue has been held with the residents.

*… I am so angry that they [the mammographic facility agents] moved it [the mammographic facility] to XX [name of the city]. But why? We have a municipality here; all those who work here pay taxes…* (Informant 7).

Additionally, some women express their insecurities regarding what to believe about the machine’s radiation effects, as well as its reliability in finding potential abnormalities.

*Well, I have mainly listened to [the media and friends] that it is supposedly dangerous, that one can get cancer from it [the mammographic screening machine].* (Informant 4).

#### Ambivalent appreciation for mammographic screening

Many of the women emphasise their appreciation for the benefit and the purpose of mammographic screening. They express their gratitude for the service of being mailed a scheduled appointment, as well as for the screening’s facilitation of an early detection of potential malignancies. However, ambivalence can occur when their understanding of the screening’s purpose conflicts with their own stance of not always accepting the invitation.

*Yes, it is a benefit because if it is [true] that we get… breast cancer, then it will be… discovered in time if one behaves and participates each [time]… one is invited.* (Informant 5)

Even though the women themselves have not participated recently, they are adamant about the importance of the participation of their relatives, as well as other women.

*… Of course, they [the daughters] should participate in mammographic screening.* (Informant 2)

There is also uncertainty regarding the personal and the professional treatment received by the women. The staff members are kind and professional, but the procedure reminds the women of an assembly line.

*… these nurses who are managing the mammographic screening are very professional and knowledgeable, and… they don’t have any viewpoints about me. “Take off your shirt,” and that is so [A: mm]. But that has nothing to do with dialogue and communication.* (Informant 6)

When some of the women call to cancel their appointments, the nurse who takes the call may or may not ask about the reason for the cancellation.

### Sense and sensibility in the decision to refrain from mammographic screening

Sense and sensibility are incorporated in the decision to undergo or refrain from the mammographic screening and are demonstrated in two generic categories: 1) physical and psychological tensions and 2) rationalisation when deciding to refrain from mammographic screening. Sense and sensibility reflect the woman’s individual reasoning when making the decision. Sense is more about the body sensations and managing the examination process, while sensibility concerns the inner, “intuitive” reasoning for not participating.

#### Physical and psychological tensions

When the women have undergone the screening, their approaches to the mammogram are linked to their distress over the mammogram and are coped with in different ways. Psychological distress over refraining from the mammogram may occur because several women perceive the invitation as something that they should accept. When they do not undergo the screening, they utter self-critical comments, such as cheating, being lazy or neglectful.

*I feel that… this is something that I, myself, have been cheating with.* (Informant 2)

The mammogram itself has both components of physical tension and psychological mobilisation in various degrees, and because a mammogram is individually experienced, it is managed in diverse ways. One approach is to relate the procedure to something else in life that is worse. Encouraging self-talk and positive thoughts are examples of other strategies.

*It [the mammogram] isn’t overly comfortable, but I mean that I will handle it exactly as… I probably have a fairly high threshold for pain.* (Informant 2)

Some of the women regard unmanageable pain and knowledge as pivotal reasons to refrain from the screening. Several women clearly state their reluctance to know if they have any malignancies. Their anxiety over being recalled and the waiting period from being examined to receiving the result are also causes for refraining from the procedure.

*… these weeks, I have thought like this, that “I will not subject myself to this again…”* (Informant 8)

This anxiety is ultimately derived from the fear of receiving the result that indicates some malignancies. There was also a reference to physical agony as another reason for non-participation.

*…they [the staff] have explained that [each] one has [a] different [level of] sensitivity.... And I have very sensitive breasts…. So, for me... it can only be compared with torture.* (Informant 10)

#### Rationalisation when deciding to refrain from mammographic screening

Many of the women display trust in themselves and express having control over their own bodies by conducting breast self-examination (BSE). The women describe trust in the awareness of one’s body and risk assessment are described by the women as being based on a strong inner conviction. A woman state:

*I am 100% convinced that I am not ill, and I will not get breast cancer.* (Informant 3)

By having this innate feeling of knowing if something is wrong in one’s body, undergoing regular mammographic screening is perceived as unnecessary.

*I am such a person who knows every little change in my body from the inside, long before anyone else can find anything wrong.* (Informant 11)

Before being interviewed, another woman asked herself why she had not undergone mammographic screening:

*Because you are absolutely convinced that the body heals itself, and [you] don’t need to attend to all the small things [that you] see.* (Informant 1)

BSE facilitates body knowledge and self-trust; it also becomes an alternative to screening. However, some women forget to conduct BSE regularly or avoid it, which then triggers their guilty conscience for not having done what they are supposed to do.

*I ought to think of these things [conducting BSE] … I am probably bad at considering those things.* (Informant 10)

Risk assessment is another factor that is considered when deciding to undergo screening or refrain from it. Some women rationalise that cancer is uncommon in their family, the usual cause of death in their family is another type of disease, and they regard their lifestyle in general as healthy.

*No one… [my] siblings or [the] women in my family... has had breast cancer. And I have [pause] lived healthily, I think, and haven’t smoked or subjected my body to hormones..., and I think that I have other health risks. I believe that it is cardiovascular [disease] that [will be the] ... cause of [my] death.* (Informant 2)

Having contracted cancer previously and undergone treatment are also perceived as forms of protection even though common sense indicates that it is not necessarily the case.

*Yes, now I have been stricken [with cancer], so now I can, now it can’t be anything else. And then I got it [cancer] again when I was 60, and then I thought that “now I am so full of cytotoxin so now I cannot get it” [again]…* (Informant 4)

Disregarding their own needs by placing those of other people before their own is common among the women. The feeling of taking something away from another individual by participating in mammographic screening has an impact on considering whether or not to undergo the procedure.

*I’m for sure not going to take another woman’s place. If I get breast cancer, then I will.* (Informant 3)

Even though refraining does not mean that someone else will have the opportunity to undergo mammographic screening, it is still one reason to refuse it. This is because the staff can then allocate more time to treatment, for instance.

### Dependency and options

The main category, “Dependency and options”, reflects structural conditions, as well as the woman’s own possibility to influence and control these circumstances. All the women stress the impact of being acknowledged as independent individuals with their own needs. Having the possibility to choose different solutions that accommodate their specific circumstances requires a responsive healthcare system.

#### Importance of flexible and individually adapted systems and solutions

Service-adapted operation and local representation, such as being invited to undergo mammographic screening, preferably in the close vicinity of the women’s places of residence, are perceived as facilitating participation. The options to undergo the procedure in the evening and on the weekend are other propositions. Some women suggest that mammographic screening could be part of another health examination, thus making it easy to participate.

*If it [the mammographic screening] is in terms of preventive healthcare, and it is located somewhere... where the majority visits now and then, [it would make it easy to just drop by for screening].* (Informant 11)

Facilitating participation by having access to a mobile unit is requested by most of the women who reside in municipalities other than the one where the only mammographic unit is located. The mobile unit was phased out around 2006 and has not been replaced, which has been a cause of frustration among the women.

*I believe that it [the mammographic facility] would have more participants if it started with the so-called bus here in XX [name of the municipality] again.* (Informant 6)

Unsatisfying individual solutions and the degree of participation are highlighted as other reasons for non-participation. If there were possibilities for more individual solutions, discussed in dialogue with the women, the prospect of participation would increase.

*… is there any possibility that I can participate in mammographic screening and get the result from a doctor immediately? “No,” they [the staff] answered. And then I asked why, and can’t one [they] have individual thinking…?* (Informant 8)

For women who refrain due to fear, another individual solution is offering them cognitive behaviour therapy to approach the screening with the support of professional coping strategies.

Some informants feel that the healthcare organisation does not listen to the women regarding potential solutions to facilitate participation. Several women report that being interviewed offers them an opportunity to be heard.

*… if my voice can make a difference regarding mammographic screening, then I will gladly participate [in the interview].* (Informant 11)

#### Insufficient possibilities to influence external circumstances and the women’s own priorities

Some determinants can either facilitate or become barriers to participation in mammographic screening. Work and time considerations influence this decision because it requires extra planning, and participation might not be prioritised.

Resource demands in relation to value include the women’s work situation, time constraints and their own health status, which all influence their decision to refrain from the screening. To a certain extent, all these factors are difficult to change, along with trying to plan around these issues because it requires more effort than what it is worth.

*… it is a threshold... that, ehhm, I am part of a system. But of course, I don’t have classes eight hours... a day, but a morning or an afternoon and sometimes a little extra. But when [pause] I get the invitation, and it is at the time when I have three hours, and I know that I must make sure [that] someone else replaces me, [it is a deterrent to accept the invitation].* (Informant 2)

It is important to make efficient and optimal use of the time spent on undergoing the screening. Many of the women residing in municipalities other than where the mammographic facility is located refer to their work situation and the importance of using their time wisely when undergoing mammographic screening, so it becomes a “kill two birds with one stone” scenario.

*… to travel to [the facility], and then it [the mammogram itself] takes such a short time, and then you have nothing to do but go home…* (Informant 9)

Prioritisation also occurs if the situation turns into a conflict between a woman’s current health status and the screening that might or might not show any malignancies. When a woman is already stricken with illness, her participation in the screening becomes subordinate.

Challenges in terms of distance and access to reliable transportation are crucial issues for some of the women, as lack of proximity to the mammographic facility require too much planning and reliance on other people. Traveling to and from the facility, knowing how to reach the hospital and considering different bus schedules also pose difficulties because the infrastructure and the logistics are in the hands of others and cause the women’s dependency.

*How shall I get there? … I can; I cannot. I don’t understand how to go by bus and... where to go and whom to meet; I cannot…* (Informant 7)

Additionally, some women express their concerns about environmental pollution. The travel distance for the women residing in other municipalities demands other means of transportation, such as driving their own vehicles that are more reliable, however taxing on the environment. In this context, the distance poses a barrier to participation and an environmental threat.

## Discussion

This study emphasises the voices of some women who have chosen to refrain from mammographic screening and contributes to a deepened understanding of the issue. Research findings need to be revisited for reconfirmation in further studies or for modifications/elaborations on future areas of investigations because times, trends, societal conditions and norms change. Instead of applying a purely biomedical approach, this discussion adopts a holistic perspective because of the complex reasons for refraining from mammographic screening. Such a standpoint also offers the benefit of being understood from different perspectives, such as socioeconomic conditions and lifestyle-related determinants of health [13]. The study also has a regional focus. To take action and influence the conditions that may negatively affect public health, a regional understanding of the health, healthcare and social determinants in the country is required. This matter is also important due to the undesired differences in health between regions and municipalities [23].

The experiences and the perceptions about mammographic screening involve doubts regarding mammographic screening and its organisation, sense and sensibility in the decision to refrain from the screening, and dependency and options. The trust and the appreciation for the opportunity to be screened and if needed, to receive treatment at an early stage, are unquestionable and corroborated by other studies [33]. However, doubts and ambivalence also exist. Mammographic screening may not detect everything [22, 29], and the fear of radiation [19, 22] makes the purpose of undergoing the screening questionable. As the technology improves, assumingly minimising the risk of radiation-induced cancers, as well as improving the test’s sensitivity and specificity, this information may pass unnoticed for the women who based their decision to refrain from mammographic screening years ago. The women also express their distrust in some decisions made by the regional healthcare organisation. The establishment is perceived as inconsiderate to the women in terms of the potentially unnecessary pain imposed on them and the inconvenient travel time of those women who do not live near the mammographic facility. These factors can be viewed as organisational barriers [34] to participation. The situation is also perceived as unequal because other regions supposedly have found more accommodating solutions. Distrust in one aspect of the healthcare organisation may spread to comprise the whole system [35] and consequently pose a threat to individual and public health since it can delay health-seeking care.

Sense and sensibility involve perceptions about the body, bodily sensations and logical reasoning. Some degree of tension and discomfort seems to occur among most of the women. Such uneasiness relates to experiences of pain, fear, having to prioritise, making decisions and not being taken seriously by the healthcare sector. These tensions may negatively affect the women’s self-assessed health, which has been linked to non-participation in mammographic screening in another study [36]. In this present study, some women’s experiences of unmanageable pain during the procedure and distress while waiting for the result of the mammogram are corroborated by previous studies’ findings [20, 22]. The fear of receiving a breast cancer diagnosis is not unrealistic because it was the leading cancer type among women in Sweden in 2016, accounting for a prevalence of 7558 women [37]. For some women, a cancer diagnosis is equal to death [38], which could be a reason to refrain from the screening as a form of coping with unmanageable knowledge. These factors are deal breakers in deciding to decline the screening invitation. However, confidence in the women’s own body knowledge and their estimated low risk of developing breast cancer can outrank the necessity to undergo the screening, which is supported by previous research [22]. The women’s own convictions, such as “not taking another woman’s place by participating in the screening,” also influence their decision to refrain from the screening. The cited conviction may be an expression of unconsciously abiding by the gender contract and an indication of “compulsive sensitivity” (caring and responding to other people’s needs before one’s own) [39] that may jeopardise the women’s actions to cater to their own health needs [40]. Consequently, the physical and the psychological resources required during the whole process of participating may be greater than the perceived value of the mammogram.

This reasoning is closely linked to the dependency and the options expressed by the women in this study. For many of these women, the dependency on others’ goodwill and decision making, as well as the lack of individual options, are deterrents to participation. The clinic hours [18] do not facilitate the participation of the working women because their employers might not approve their taking time off for the screening. For the women themselves, their commitment to work and potentially having to find a substitute or to reschedule work appointments are deterrents and potential stressors. Work stress with time pressure has increased in most of the socioeconomic groups in Sweden since the 1990s [41], and the estimated “cost” of undergoing the screening in relation to the women’s work situation is higher than the perceived value of the benefit.

Additionally, being unable to influence when to participate in the screening, having to rely on public transportation or others’ goodwill (when knowing about the previously existing mobile unit that addressed the need for proximity) and not being offered individual solutions are causes of the women’s frustration and grief. The studies conducted to understand non-participation in mammographic screening have to a large extent found similar reasons for the decision to refrain from the procedure [14, 17–22, 42], and this present study is no exception.

In this study, the women have all undergone mammographic screening on one or more occasions. Research has shown a higher likelihood of participation if the women have previously undergone the procedure [11]; thus, it could be assumed that the decision to participate or decline the invitation can be changed and be a target for interventions. This study contributes to identifying potential threats to continuous participation, which could be assumed as important because activities that entail too many barriers to overcome are less stable [43]. A participant may become a non-participant. A previous study conducted in the same region as this present one, but involving women who participated in mammographic screening and described similar structural and individual threats to participation [44], offers even more support for the determinants of interest to secure future screening participation.

This present study’s findings can be reflected on in relation to the concept of access [45], where the wish for a mammographic facility in the vicinity is articulated and refers to the accessibility dimension. This issue is vital for the participation in mammographic screening and is supported by previous studies [14–16, 42, 46]. The impact of service mindedness is also mentioned by the women and corresponds to the accommodation dimension [45], which should not be overlooked because it serves as either a facilitator of or a barrier to participation in the screening. It is also essential to understand the complexity of undergoing mammographic screening in particular and by extension, healthcare in general. Access occurs in the interface between individual characteristics and social circumstances, on one hand, and healthcare, on the other hand [47], which in turn also regulate the degree of participation.

The women in this study all express some forms of unmet needs. Some women clearly articulate their desire to be acknowledged and perceived as persons, with lives outside healthcare, in their own contexts and as members of society with norms that can inhibit their own health behaviours. Their individual prerequisites require the healthcare staff to listen and engage in a more patient-participating relationship. Many of the women report their unfulfilled wish to participate in the screening, but their decision to refrain from the procedure is a consequence of the unresponsiveness of a healthcare provider.

This issue warrants both individual and collective patient-participation care [48], allowing such an approach to influence the women’s own healthcare preferences, as well as the health services. Consequently, it contributes to integrated people-centred care [49], which strives to offer a healthcare system based on a holistic understanding of the determinants that affect public health.

## Limitations and strengths

It was challenging to recruit the non-participating women for an interview concerning their experiences and perceptions about mammographic screening. It could be that those women did not want to validate their reasons for this decision in an interview or that the topic was not of interest to them at all. The action of not participating in mammographic screening may be a statement itself. Eleven of the 200 women who were asked to participate were interviewed in this study, which could be perceived as a limitation; however, as this is a qualitative study, its goal is not to generalise the findings to a larger population. Nonetheless, some of this study’s findings are corroborated by those of previous studies about non-participation in mammographic screening and could allow the findings to be transferred to a similar context. The transferability [50] is for the reader to reflect on. Regarding the women’s age distribution, no one was in her early 40s, which could have added another perspective. It can be perceived as a strength that all the women had undergone mammographic screening at least once although they subsequently refrained from it, as it is important to understand that decisions are not static. The regional focus may be of interest for other regions with the same set-up regarding resource allocation from the government to the regional level and concerned with maintaining or increasing the rate of participation in mammographic screening. Only one woman came from another country, which is a potential limitation because all perspectives enhance the understanding of the reasons for non-participation.

## Conclusions

The women in this study had all previously participated in mammographic screening but subsequently chose to refrain from it for several reasons. This finding indicates that decisions can be reversed if the conditions change. This provides positive knowledge for the healthcare organisation if it wants to increase the women’s participation rate. There is a need for the women to be involved in the healthcare decisions concerning themselves, not merely remain as passive recipients of healthcare. Participation requires trust and can be achieved by listening to the citizens’ voices. Understanding these women’s experiences and perceptions may also be valuable for public participation in similar services. By considering the experiences of the women, better access to health services can be gained.

## Supplementary information

**Additional file 1.** Interview guide

## Abbreviations

[A]: Author
“A”: Actively passive
“D”: Attended in mammographic screening
“P”: Passive active
BSE: Breast self-examination
COREQ: Consolidated criteria for reporting qualitative studies

## References

[1] World Health Organization. The Ottawa Charter. Health Promotion. 1986;1(4):iii-v.

[2] Frenk J (2004). Health and the economy: a vital relationship. Organisation for economic cooperation and development. The OECD Observer.

[3] Donabedian A (1972). Models for organizing the delivery of personal health services and criteria for evaluating them. The Milbank Memorial Fund Quarterly.

[4] Socialstyrelsen [National Board of Health and Welfare] (2014). Screening för bröstcancer – Rekommendation och bedömningsunderlag. Screening for breast cancer – Recommendations and basis for assessment.

[5] Wilson JMG, Jungner G (1968). Principles and practice of screening for disease.

[6] Vainio H, Bianchini F (2002). IARC handbooks of cancer prevention. Breast cancer screening.

[7] Morabia A, Zhang F (2004). History of medical screening: from concepts to action. Postgrad Med J.

[8] Duffy S, Chen T, Smith R, Yen A, Tabar LR (2013). artificial controversies in breast cancer screening. (report). Breast Cancer Manag.

[9] Puliti D, Duffy SW, Miccinesi G, De Koning H, Lynge E, Zappa M, Paci E (2012). Overdiagnosis in mammographic screening for breast cancer in Europe: a literature review. J Med Screen.

[10] Independent UK Panel on Breast Cancer Screening (2012). The benefits and harms of breast cancer screening: an independent review. Lancet.

[11] Bankhead CR, Brett J, Bukach C, Webster P, Stewart-Brown S, Munafo M, Austoker J (2003). The impact of screening on future health-promoting behaviours and health beliefs: a systematic review. Health Technol Assess.

[12] Statens Offentliga Utredningar [Official Reports of the Swedish Government] (2009). En nationell cancerstrategi för framtiden [A national cancer strategy for the future].

[13] Dahlgren G, Whitehead M (2007). European strategies for tackling social inequities in health: levelling up, part 2.

[14] Zidar MN, Larm P, Tillgren P, Akhavan S (2015). Non-attendance of mammographic screening: the roles of age and municipality in a population-based Swedish sample. Int J Equity Health.

[15] Jewett PI, Gangnon RE, Elkin E, Hampton JM, Jacobs EA, Malecki K, LaGro J, Newcomb PA, Trentham-Dietz A (2018). Geographic access to mammography facilities and frequency of mammography screening. Ann Epidemiol.

[16] Huang B, Dignan M, Han D, Johnson O (2009). Does distance matter? Distance to mammography facilities and stage at diagnosis of breast cancer in Kentucky. J Rural Health.

[17] Jensen Flytkjaer L, Pedersen A, Andersen B, Fenger-Grøn M, Vedsted P (2013). Distance to screening site and non-participation in screening for breast cancer: a population-based study. J Public Health.

[18] Fallowfield L, Rodway A, Baum M (1990). What are the psychological factors influencing attendance, non-attendance and re-attendance at a breast screening Centre?. J R Soc Med.

[19] Watson-Johnson LC, DeGroff A, Steele CB, Revels M, Smith JL, Justen E, Barron-Simpson R, Sanders L, Richardson LC (2011). Mammography adherence: a qualitative study. J Women's Health.

[20] Sterlingova T, Lundén M (2018). Why do women refrain from mammography screening?. Radiography.

[21] Johansson I, Berterö CM (2003). Getting no respect: barriers to mammography for a group of Swedish women. Health Care Women Int..

[22] Lagerlund M, Widmark C, Lambe M, Tishelman C (2001). Rationales for attending or not attending mammography screening – a focus group study among women in Sweden. Eur J Cancer Prev.

[23] Kommissionen för jämlik hälsa [The Swedish Commission for Equity in Health] (2017). Nästa steg på vägen mot en mer jämlik hälsa. Slutbetänkande av Kommissionen för jämlik hälsa The next step towards more equity in health. Final report by the Swedish Commission for Equity in Health. SOU 2017:47.

[24] Edwards R, Holland J (2013). What is qualitative interviewing?.

[25] Statistiska Centralbyrån [Statistics Sweden]. Folkmängden efter region, civilstånd, ålder och kön. År 1968–2018. [Population by region, marital status, age and sex. Year 1968–2018]. Sweden: Statistiska Centralbyrån [Statistics Sweden]; 2020. http://www.statistikdatabasen.scb.se/pxweb/sv/ssd/START__BE__BE0101__BE0101A/BefolkningNy/. Accessed 8 Jan 2020.

[26] OECD/European Observatory on Health Systems and Policies. Sweden: Country health profile 2019, State of Health in the EU. Brussels: OECD Publishing, Paris/European Observatory on Health Systems and Policies; 2019. https://doi.org/10.1787/2dcb7ca6-en.

[27] Cancerfonden [Swedish Cancer Society] (2015). Cancerfondens kartläggning för år 2015 [Swedish Cancer Society’s survey for 2015]. Unpublished work.

[28] Polit DF, Beck CT (2010). Essentials of nursing research: appraising evidence for nursing practice.

[29] Manjer ÅR, Zackrisson S, Emilsson UM (2016). On women’s ambivalence about mammography screening: support in the decision-making process a potential role for health care social workers?. Br J Soc Work.

[30] Krueger RA, Casey MA (2015). Focus groups: a practical guide for applied research.

[31] Elo S, Kyngäs H (2008). The qualitative content analysis process. J Adv Nurs.

[32] Tong A, Sainsbury P, Craig J (2007). Consolidated criteria for reporting qualitative research (COREQ): a 32-item checklist for interviews and focus groups. Int J Qual Health Care.

[33] Manjer ÅR, Emilsson UM, Zackrisson S (2015). Non-attendance in mammography screening and women’s social network: a cohort study on the influence of family composition, social support, attitudes and cancer in close relations. World J Surg Oncol.

[34] Gulliford Martin (2013). Access to Health Care.

[35] LaVeist TA, Isaac LA, Williams KP (2009). Mistrust of health care organizations is associated with underutilization of health services. Health Serv Res.

[36] Jensen Flytkjaer L, Pedersen A, Andersen B, Vedsted P (2015). Self-assessed health, perceived stress and non-participation in breast cancer screening: a Danish cohort study. Prev Med.

[37] Johansson E (2018). Cancer i siffror 2018 [Cancer in figures 2018].

[38] Powe BD, Finnie R (2003). Cancer fatalism: the state of the science. Cancer Nurs.

[39] Forssén A, Carlstedt G, Mörtberg C (2005). Compulsive sensitivity—a consequence of caring: a qualitative investigation into women carer’s difficulties in limiting their labours. Health Care Women Int.

[40] Sisk RJ (2000). Caregiver burden and health promotion. Int J Nurs Stud.

[41] Hemström Örjan (2001). Chapter 7. Working Conditions, the Work Environment and Health. Scandinavian Journal of Public Health.

[42] Brustrom JE, Hunter DC (2001). Going the distance: how far will women travel to undergo free mammography?. Milit Med.

[43] Mechanic D (1999). Issues in promoting health. Soc Sci Med.

[44] Norfjord Van Zyl M, Akhavan S, Tillgren P, Asp M (2018). Experiences and perceptions about undergoing mammographic screening: a qualitative study involving women from a county in Sweden. Int. J. Qual. Stud. Health Well-being.

[45] Penchansky R, Thomas JW (1981). The concept of access: definition and relationship to consumer satisfaction. Med Care.

[46] Jensen LF, Pedersen AF, Andersen B, Fenger-Gron M, Vedsted P (2014). Distance to screening site and non-participation in screening for breast cancer: a population-based study. J Public Health.

[47] Mandelblatt JS, Yabroff KR, Kerner JF (1999). Equitable access to cancer services: a review of barriers to quality care. Cancer: Interdisciplinary International Journal of the American Cancer Society.

[48] Castro EM, Van Regenmortel T, Vanhaecht K, Sermeus W, Van Hecke A (2016). Patient empowerment, patient participation and patient-centeredness in hospital care: a concept analysis based on a literature review. Patient Educ Couns.

[49] Framework on integrated, people-centred health services. Report by the Secretariat. A69/39. http://apps.who.int/gb/ebwha/pdf_files/WHA69/A69_39-en.pdf?ua=1. Accessed 16 Jan 2020.

[50] Graneheim UH, Lundman B (2004). Qualitative content analysis in nursing research: concepts, procedures and measures to achieve trustworthiness. Nurse Educ Today.

[51] World Medical Association. World Medical Association Declaration of Helsinki. Ethical principles for medical research involving human subjects. Bull. World Health Organ. 2001;79(4):373. PMC2566407 11357217

