# Supplementary material for: Non-participation in mammographic screening – experiences of women from a region in Sweden
Source: BMC Public Health. 2020 Feb 12;20:219. doi: 10.1186/s12889-020-8306-8 (PMC7017469; doi:10.1186/s12889-020-8306-8)
Supplement: Supplementary file 1 — Additional file 1. Interview guide [file 12889_2020_8306_MOESM1_ESM.docx]

**Additional file 1 Interview Guide
Opening Question**

Can you tell me about yourself?

**Introductory Question**

What do you think of when you hear the word mammogram?

**Transition Question**

Try to remember when you first received the invitation to mammographic screening. What did you feel and think?

**Key Questions**

How did you reason around your decision to refrain from mammographic screening?

What factors have influenced your decision to refrain?

What do you think is the most important reason for you to decline the invitation?

What advice would you give to a friend or a family member regarding participating in mammographic screening?

What do you think about the information that is provided about mammographic screening?
(from the healthcare system and society)

Have you in any other way engaged with the healthcare system? (when, where, etc.)
How do you feel about the healthcare system in general?

**Ending Question**

Based on what we have discussed – if you could decide, what would you change in order to increase women’s participation in mammographic screening?

Of what we have discussed – what do you think is the most important and why?

Why did you agree to participate in this interview?

**Summary Question***The interviewer presents a summary of what has been said during the interview.*Do you agree with this summary of the interview?

**Final Question**Is there anything you would want to change or add?
